# Comprehensive machine learning based study of the chemical space of herbicides

**DOI:** 10.1038/s41598-021-90690-w

**Published:** 2021-06-01

**Authors:** Davor Oršolić, Vesna Pehar, Tomislav Šmuc, Višnja Stepanić

**Affiliations:** 1 Laboratory for Machine Learning and Knowledge Representation, Division of Electronics, Ruđer Bošković Institute, Bijenička 54, 10002 Zagreb, Croatia; 2 Croatian Defense Academy “Dr. Franjo Tuđman“, Ilica 256b, 10000 Zagreb, Croatia

**Keywords:** Plant sciences, Cheminformatics

## Abstract

Widespread use of herbicides results in the global increase in weed resistance. The rotational use of herbicides according to their modes of action (MoAs) and discovery of novel phytotoxic molecules are the two strategies used against the weed resistance. Herein, Random Forest modeling was used to build predictive models and establish comprehensive characterization of structure–activity relationships underlying herbicide classifications according to their MoAs and weed selectivity. By combining the predictive models with herbicide-likeness rules defined by selected molecular features (numbers of H-bond acceptors and donors, logP, topological and relative polar surface area, and net charge), the virtual stepwise screening platform is proposed for characterization of small weight molecules for their phytotoxic properties. The screening cascade was applied on the data set of phytotoxic natural products. The obtained results may be valuable for refinement of herbicide rotational program as well as for discovery of novel herbicides primarily among natural products as a source for molecules of novel structures and novel modes of action and translocation profiles as compared with the synthetic compounds.

## Introduction

Herbicides are compounds of small molecular weight used for selective destruction of weeds. Because of their extensive use, the two global issues have appeared in the last two decades, an increase in weed resistance and health issues^1^. In order to circumvent development of weed resistance, herbicides with different modes of action (MoAs) are applied rotationally. Herbicides are classified according to the MoAs in ~ 25 classes within the two similar classification systems—HRAC and WSSA, set up by Herbicide Resistance Action Committee of Australia and Weed Science Society of America, respectively^2–5^. The MoAs denote the biochemical processes in weeds which herbicides modify (Table 1). Given the common name of a herbicide, the classification schemes in addition to MoA also provide the chemical family a herbicide belongs to. Sub-classification to the chemical families according to possessing common fragment(s) was made in order to refine herbicide rotation scheme and increase its efficiency against the weed resistance. The chemical sub-classification of the herbicides is, however, not unequivocal. Different number of chemical sub-groups have been defined in the HRAC and WSSA systems and recently by Forouzesh^6^.

**Table 1:** HRAC classification and division of herbicides from the HRAC2020 and extended data sets across the MoA classes^a^.

Legacy hrac code	hrac2020&wssa code	Number of compounds in hrac2020/extended set	General mode of action–targeted biological process	Mode of action–targeted molecular functions
A	1	21/29	Fatty acid biosynthesis	Inhibition of acetyl-CoA carboxylase (ACCase)
B	2	58/61	Amino acid synthesis (Leu, Ile, Val)	Inhibition of acetohydroxyacid synthase/acetolactate synthase (AHAS/ALS)
C1	5	43/53	Photosynthesis (electron transfer)	Inhibition of photosystem (PS) II protein D1 (C1/C2 Ser264; C3 His215)
C2	5	30/37		
C3	6	5/9		
D	22	4/5	Photosynthesis (electron transfer)	Inhibition of diversion of the electrons transferred by the PS I ferredoxin
E	14	29/43	Photosynthesis (heme synthesis for chlorophyll)	Inhibition of protoporphyrinogen oxidase (PPO)
F1	12	7/9	Photosynthesis (carotenoid synthesis)	Inhibition of phytoene desaturase (PDS)
F2	27	14/16		Inhibition of 4-hydroxyphenylpyruvate dioxygenase (4-HPPD)
F3	34	1/2		Inhibition of lycopene cyclase
F4	13	2/1		Inhibition of 1-deoxy-d-xylulose-5-phosphate (DOXP) synthase
G	9	1/2	Amino acid synthesis (Phe, Trp, Tyr)	Inhibition of 5-enolpyruvylshikimate-3-phosphate (EPSP) synthase
H	10	2/4	Amino acid synthesis (Gln)	Inhibition of glutamine synthase
I	18	1/3	Tetrahydrofolate synthesis	Inhibition of dihydropteroate (DHP) synthase
K1	3	18/25	Microtubule polymerization	Inhibition of microtubule assembly
K2	23	6/9		Inhibition of microtubule organisation
K3	15	43/39^a^	Fatty acid synthesis	Inhibition of VLCFAs
L	29^b^	6/6	Cell wall synthesis	Inhibition of cellulose synthase
M	24	6/8	ATP synthesis	Uncoupling of oxidative phosphorylation
N	NA^b^	NA^b^/23	Fatty acid synthesis	Inhibition of fatty acid elongase
O	4	25/37	Regulation of auxin-responsive genes	Synthetic auxin mimics -Stimulation of transport inhibitor response protein 1 (TIR1)
P	19	2/3	Long-range hormone signaling	Auxin transport inhibitors

^a^In the HRAC2020 classification there are additional classes Q (3), R (31), S (32) and T (33), all with up to 2 members ^5^.

^b^Majority of herbicides from the class N are fused in the K3 (15) class. The treating 23 herbicides of the legacy N class separately, does not affect the results since this subgroup is structurally diverse from the other K3 herbicides.

Among the MoAs, ten of them are identified with the inhibition of specific enzymes and are associated by around half of the used herbicides (Table 1). However, the precise mechanisms of action of herbicides resulting in their phytotoxic effects are rarely known ^7^. For example, herbicides from the most populated and used class B are all inhibitors of the enzyme acetolactate synthase (ALS), known also as acetohydroxyacid synthase (AHAS), which catalyzes the first step in the synthesis of the branched-chain amino acids valine, leucine, and isoleucine. However, their phenotypic inhibitory effects can be different what may be due to different binding modes onto ALS/AHAS and/or their different translocation properties through weeds^7,8^. Herbicides of different MoAs have also different propensities to induce weed resistance because of not only different prevalence of their usage, but also different sites of action (SoAs) and translocation properties.

The MoA classification schemes for herbicides are examples of the application of the structure–activity relationship (SAR) analysis. The general SAR assumption is that structurally similar compounds share SoA. The sub-partition of MoA classes into chemical families is in the line with this assumption. However, such an assumption does not imply that compounds which are structurally dissimilar may not have the same SoA/MoA what may afflict the usage of the classification schemes in the rotational anti-resistance strategy. Indeed, it has been demonstrated by scaffold hopping methods in design of novel biologically active compounds that dissimilar structures can have the same MoA^9^. Furthermore, there is an open question how much compounds belonging to different MoA classes are mutually structurally similar and may hence act in similar way what can also impair the rotational strategy.

The other approach to circumvent weed resistance is through discovery of novel molecules with different MoA. The valuable source of such molecules is natural products (NPs)^10^. The first of the two main objectives of our computational study was to provide a formal rationale for the underlying SAR assumption of the MoA classification schemes used in confrontation with the worldwide increase in the weed resistance and to point out potential limitations of MoA labelling with using only structural similarity. In an attempt to improve herbicide characterization and thus rotational strategy, categorizations of herbicides according to their application stage and weed selectivity were also modelled for the first time as far as we are aware. By combining machine learning (ML) models with a set of herbicide-likeness rules, virtual screening platform is proposed. Another objective was to enrich the phytotoxic chemical space with molecules having novel MoA. For this purpose, the screening cascade was applied on the set of phytotoxic NPs.

## Methods

### Data sets

The calculations were done with the data set HRAC2020 of 346 mainly synthetic organic herbicides downloaded from the original HRAC list and its extended version of 509 herbicides with relative molecular weight within the range 84–649^5^. The extended data set contains additional 163 mostly obsolete herbicides collected from the literature and open-source online databases: Compendium of Pesticide Common Names (http://www.alanwood.net/pesticides/), PPDB: Pesticide Properties Database, PubChem and PTID: Pesticide Target Interaction Database^6,11–13^. The MoAs were assigned for 411 compounds according to the legacy HRAC system (314 herbicides from the HRAC2020 set) and on the basis of belonging to chemical families (97 herbicides forming the subset HRAC_REST) (Table 1)^5,6,14^. The remaining 98 herbicides herein referred as the Z class, were unclassified (Supplementary Table S1). The data on application stage and weed selectivity were collected for subsets of 221 and 332 herbicides, respectively^14^. The data set of 131 phytotoxic NPs was collected from literature (Table S2) ^15–24^.

### Molecular descriptors

The cleaned SMILES were used as inputs for the calculations of 1D and 2D molecular descriptors by the R package *rcdk*^25^ and the programs DataWarrior^26^ and ADMET Predictor 9.5 (Simulations Plus, Inc., USA)^27^. The *rcdk* descriptors were structural fingerprints (fp) (11 different types including extended and 166-bit MACCS fps), constitutional (17 of them), electronic (6) as well as hybrid BCUT (6) descriptors. The 141 MACCS keys which were present in more than five herbicides were used as descriptors. Physicochemical and simple structural properties which govern uptake and translocation properties of herbicides through plants^28–34^ were calculated by DataWarrior (27) and ADMET Predictor 9.5 (139). The net ionization state of molecules was roughly estimated as a difference of numbers of basic nitrogen (pKa above 7.0) and acidic oxygen atoms (pKa below 7.0) calculated by DataWarrior. Prior to modelling, descriptors (except fp) were scaled as (x − mean(x))/sd(x).

### Hierarchical clustering

Hierarchical clustering was performed with wardD.2 minimum variance agglomeration method and Tanimoto coefficient (TC) as a similarity index by the stratified sampling function *hclust*. The Dunn (the ratio: the cluster minimum separation/the maximum cluster diameter) and Dunn2 (the minimum average dissimilarity between two clusters/the maximum average dissimilarity within cluster) indices as well as average Silhouette (Si) width (compares the average distance to elements in the same cluster with the average distance to elements in other clusters) were used for internal clustering validation. The adjusted Rand index (ARI) was applied in order to assess the similarity of the predicted grouping with the legacy HRAC labels. The three internal validation scores are higher and better when clusters are dense and well separated. Considering external validation, more similar groupings has a positive ARI closer to 1. The clustering validation indices were calculated by the R package *fpc*.

### Modelling

The multi-classification modeling in terms of subsets of various kinds of descriptors was performed by Random Forest (RF) method ('rf') available in the R package *caret* with one tunable parameter (mtry, a number of variables randomly sampled at each split) and using tenfold cross-validation (CV). The HRAC classes with less than 3 members (Table 1) were excluded from modelling and these compounds were added to the Z class. The remaining 314/419 compounds from the HRAC2020/extended set were divided into training and test sets in the 80:20 ratio, except in the case of the classes with 3–5 members, for which 50:50 ratio was applied. The splitting was done using stratified random sampling. Thus, in the case of original/ extended herbicide set, there were 257/341 training and 57/78 test compounds arranged in 16/19 classes. Analogous dividing procedure was applied for the subsets of 221/332 compounds with assigned application stage/weed selectivity.

Further, in order to optimize performance of MoA and weed selectivity models in terms of selected descriptors, the hyperparameter tuning of RF and three additional classifiers eXtreme Gradient Boosting (XGBoost), support vector machines (SVM, RBF kernel) and naive Bayes (NB) as a baseline model, all available in *caret*, were carried out by using grid search and 10 runs of tenfold CV as well as by keeping all resamples for performance comparison (Figures S1−S4). For RF and NB classifiers, parameter tuning was done by utilization of the packages *randomForest* and *klaR*, respectively. The final models were built with optimal values of tuning parameters on the entire training HRAC2020 set. The classifiers were compared mutually by analyzing resampling distributions and using Bayesian analysis (Python library *baycomp*)^35^ as well as by their performance on the test test.

The model predictive capacity was assessed by counting the numbers of true positives (TP), true negatives (TN), false positives (FP), and false negatives (FN) for each class and usage of following performance metrics: sensitivity (Sensitivity or Recall = TP/(TP + FN)), precision ( Precision = TP/(TP + FP)), specificity (Specificity = TN/(TN + FP)), overall predictive accuracy (Accuracy = (TP + TN)/(TP + FP + FN + TN)), F1 score (F1 Score = 2*(Recall * Precision)/(Recall + Precision)) and Cohen’s unweighted kappa (Cohen's kappa = (Po − Pe)/(1 − Pe), where observed probability is Po = (TP + TN)/(TP + TN + FP + FN), and probability by chance is Pe = ((TP + FN) * (TP + FP) + (FP + TN) * (FN + TN))/(TP + TN + FP + FN)^2).

### Applicability domain (AD)

ADs were defined in terms of similarity with training compounds and the class probability outputs from the RF models^36^. Structural similarity between two molecules was estimated by using 141 MACCS keys and the coefficient TC as a similarity measure. Similarity in physicochemical space is assessed by applying the Euclidian distance.

### Violin and PCA plots

The violin plots with relevant statistical details for comparison subgroups of herbicides in molecular properties were made by using the *ggstatsplot*. The principal component analysis (PCA) was done with *princomp*.

The R computing was done within RStudio (R version 3.6.3) environment^37^.

## Results and discussion

### HRAC classification—descriptor and model selection

The multi- classification of herbicides according to MoAs in terms of subsets of various kinds of molecular descriptors was performed by RF modelling. The results obtained for the HRAC2020 and extended data sets were consistent. The best classification performance for the extended test set was obtained by using MACCS keys as molecular descriptors (Table S3). With other kinds of descriptors, the models somewhat deteriorated most probably because they do not contain information on specific structural arrangements of atoms within molecules. The constitutional descriptors (e.g. MW, numbers of atoms in the longest aliphatic chain, the largest pi system or of aromatic atoms), lipophilicity parameter and electronic descriptors (e.g. topological polar surface area (TPSA), numbers of hydrogen bond acceptor (HBA) or donor (HBD) atoms, molecular atomic and bond polarizabilities) are more general and global molecular characteristics whose values do not correlate with structural arrangement. The hybrid BCUT descriptors were also not efficient as MACCS fp in differentiation of herbicides with different MoAs although they are known for their usefulness in description of chemical diversity^38^. The MACCS structural keys better represent the scaffolds characterizing the chemical series of herbicides than other explored fp types.

**Figure 1:**
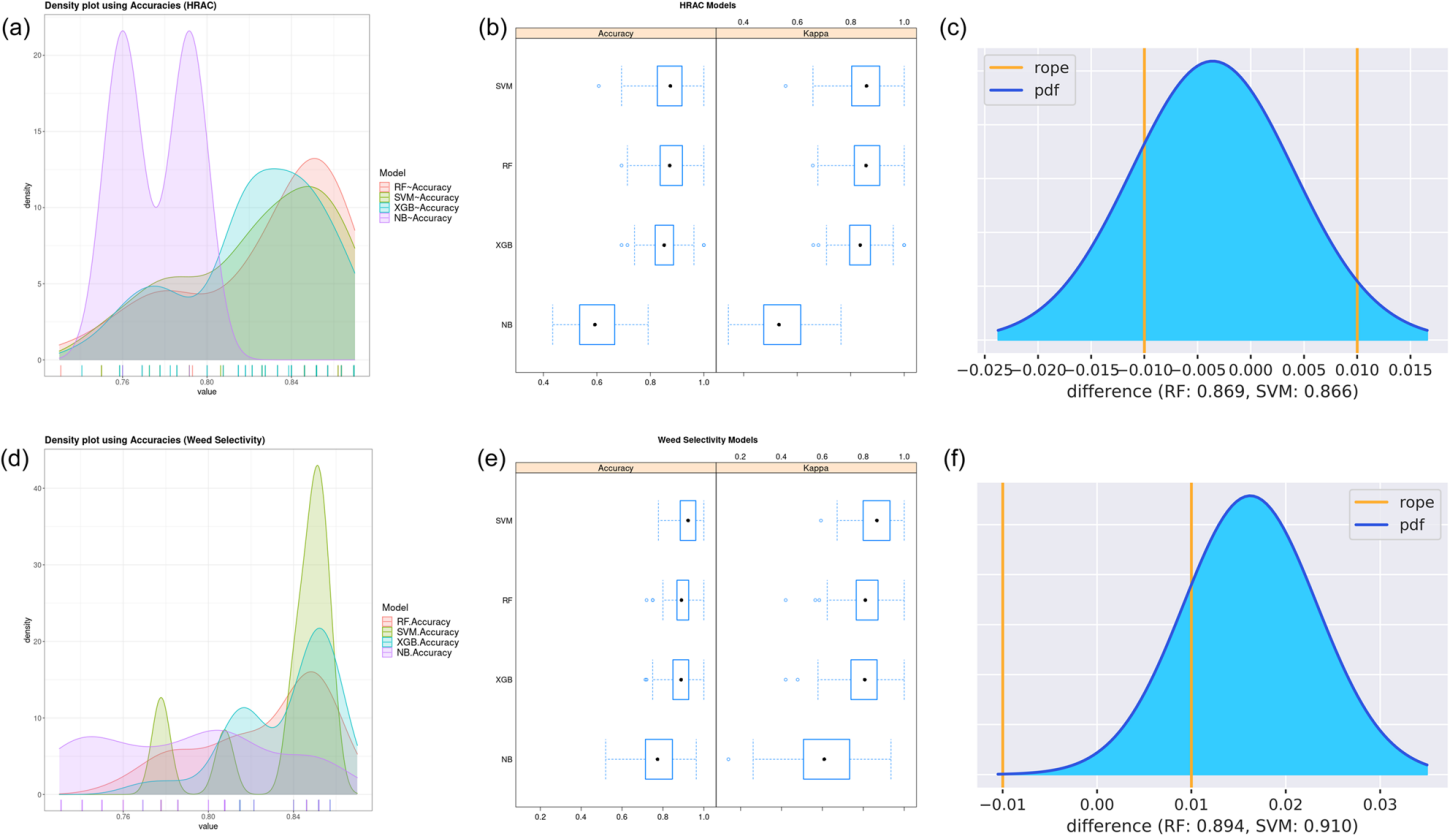
Comparing performance of the four ML classifiers for MoA predictions. (**a**, **d**) Density accuracy plot. (**b**, **e**) Box plots of distributions of resampled accuracies and kappa values. (**c**, **f**) Probability density plot for accuracy differences between the RF and SVM classifiers. The plots (**a**)–(**c**) described MoA classifiers (Table 2) and those (**d**)–(**f**) present comparison of weed selectivity models built with nine descriptors including log P (Table 3). The RF and SVM MoA classifiers are largely equivalent since 75.7% of posterior probability distribution is inside the region of practical equivalence (rope, the differences of accuracy are less than 1%).

The performance of the RF model was optimized by hyperparameter tuning along with 10 times tenfold CV resampling. The three additional ML classifiers XGBoost, SVM and NB were also explored and tuned in analogous way using the same seed to secure that folds between models contain the same set of compounds (Figure S1, Table S4). The Bayesian analysis for comparing performances of multiple classifier showed that RF and SVM(RBF) exhibit similar performance on the HRAC problem, dominating XGBoost while NB was clearly outperformed by the rest (Fig. 1a–c). The outputs of the RF and SVM (Table 2, Table S1) as well as MACCS keys determined as important (Table S5) for 16-class MoA categorization by both ML approaches are largely equivalent. They differ in predictions for 5 test and 12 HRAC_REST case compounds, which were all predicted with the RF class probabilities less than the cut-off value (see further).

**Table 2:** Comparison of classification performance on the test and HRAC_REST case sets of the four optimized 16- class MoA ML models built in terms of 141 MACCS fp keys^a^.

MoA	Overall^b^		Averaged across classes				
Classifier	Accuracy	Kappa	Sensitivity	Specificity	Precision	F1	Balanced Accuracy
**TEST SET**							
RF	0.895	0.883	0.821	0.993	0.896	0.900	0.907
XGBoost	0.895	0.883	0.821	0.993	0.899	0.899	0.907
SVM	0.912	0.902	0.838	0.994	0.935	0.936	0.916
NB	0.561	0.500	0.332	0.969	0.663	0.604	0.651
**HRAC_REST SET**							
RF	0.674	0.646	0.641	0.979	0.728	0.796	0.814
XGBoost	0.663	0.633	0.594	0.978	0.670	0.771	0.790
SVM	0.696	0.667	0.631	0.980	0.673	0.797	0.809
NB	0.413	0.362	0.310	0.961	0.509	0.605	0.638

^a^Optimal values of classifiers’ hyperparameters are listed in Table S4.

^b^The overall accuracy and kappa values are averaged over 10 × 10-fold CV resamplings.

Although SVM slightly overperformed the RF model (Table 2), we decided to perform further analysis with the RF outputs. The primary reason was possibility to use direct RF output class probabilities for definition of the model’s AD. Using SVM in the context of AD definition would require additional calibration of the SVM scores, to turn them into probabilities^39^.

### HRAC classification and structural similarity—Chemical space analysis

The classification of herbicides into the HRAC/WSSA classes (Table 1) facilitate the rotational use of herbicides of different MoA as a strategy against the weed resistance^5^. To the best of our knowledge, the sub-classification into chemical families has been done by visual inspection^6^. Herein by applying ML approaches it is shown in an objective, formal way that dividing herbicides into chemical families and also MoA classes is based on their structural similarity.

Regardless of used descriptors (Table S3) and ML algorithm (Table 2), the MoA models were generally characterized with the higher specificity than sensitivity averaged across the classes. Such a performance points to a degree of similarity between the herbicides designated to different classes what is also supported by the clustering analysis. The herbicides were clustered primarily according to common scaffolds.

**Figure 2:**
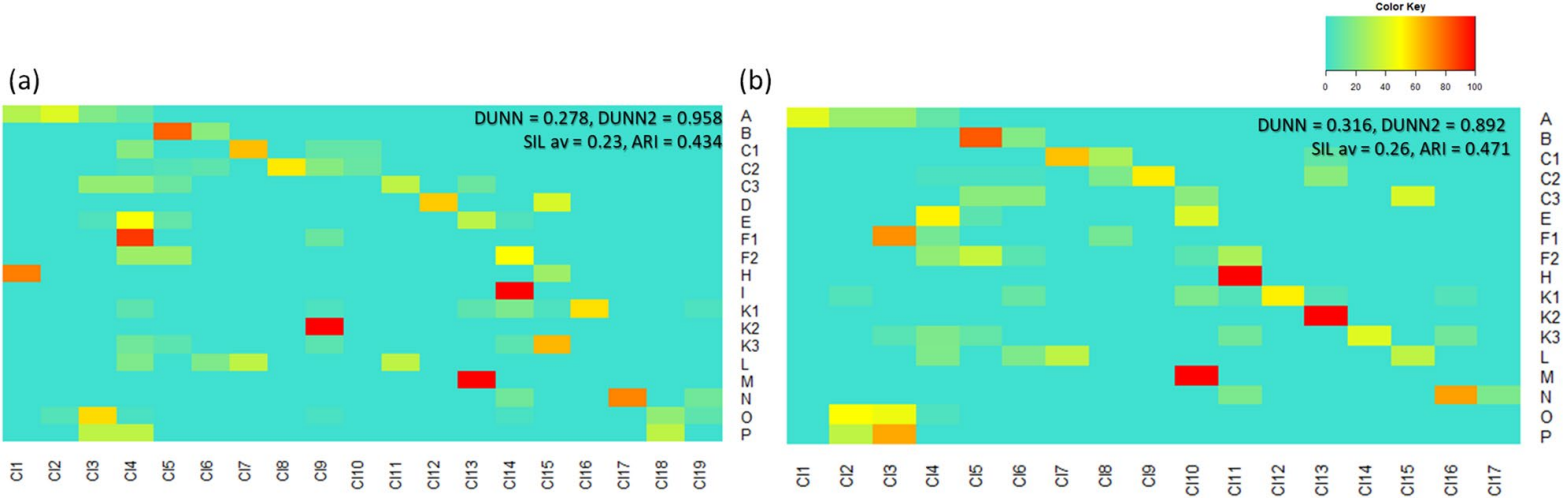
Heat map presentations and evaluation metrics for distributions of (**a**) HRAC2020 + HRAC_REST (411) and (**b**) HRAC2020 (314) herbicides in terms of fractions (%) of MoA classes in clusters generated by the agglomerative algorithm and MACCS fp.

**Figure 3:**
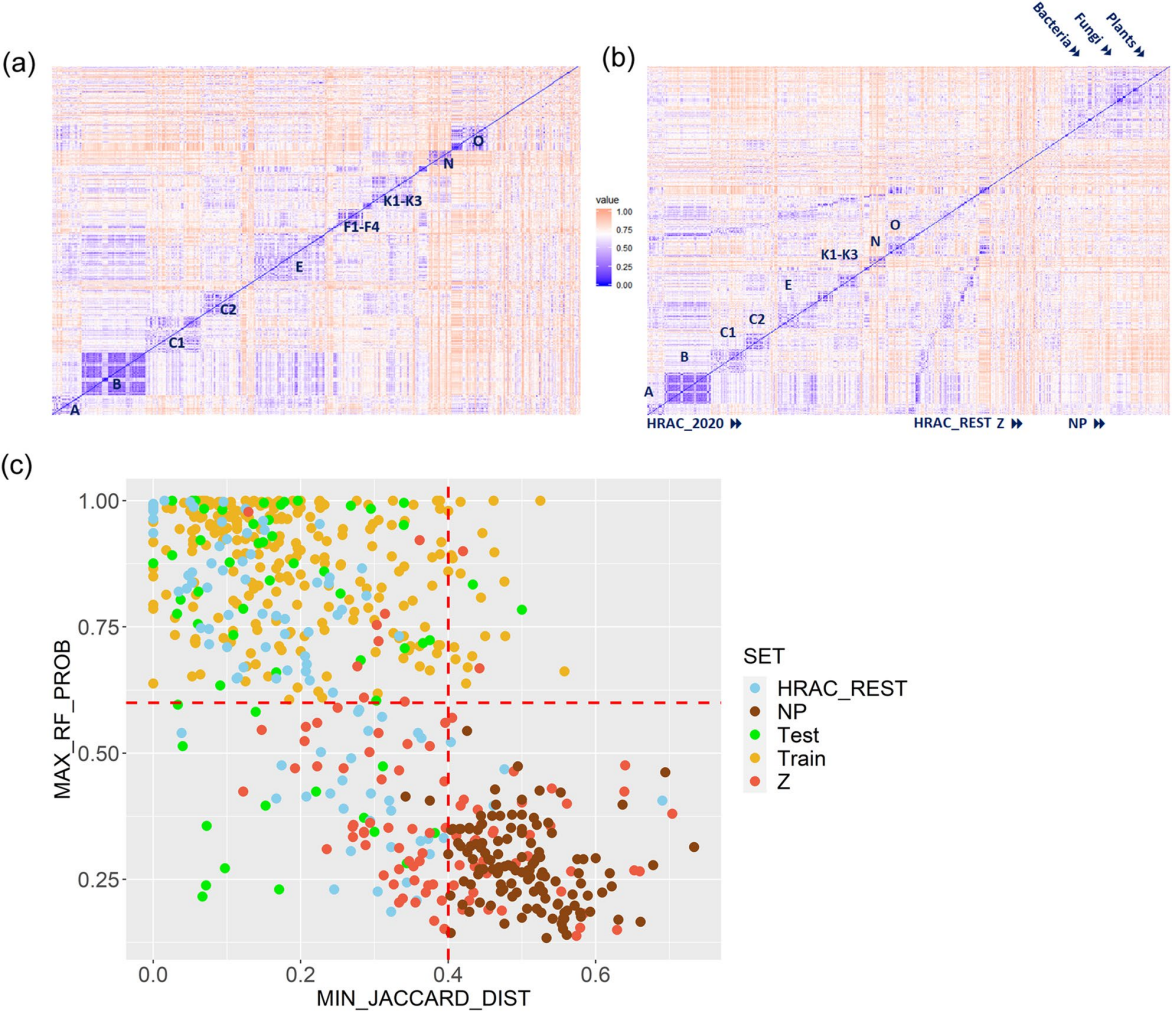
Heat maps for structural dissimilarity quantified by Jaccard coefficient(1-TC) calculated for all pairs of 509 synthetic herbicides (**a**) arranged into MoA classes and (**b**) divided into the subsets HRAC2020, HRAC_REST and the Z compounds with addition of the set of NPs originated from bacteria, fungi and plants. The extended, HRAC2020 and HRAC_REST compounds are ordered according to the classes A-P. More blue/red values correspond to more structurally similar/diverse compounds. (**c**) Definition of AD for the RF MoA model (Table 2): given a compound, the model’s prediction is considered reliable if it is similar to at least one training herbicide with TC greater than 0.6 and the estimated class probability is greater than 0.6.

This resulted in only moderate value of ARI index signifying relatively weak agreement between the generated clusters and the HRAC classes (Fig. 2). The inter-cluster distances were also described by relatively low values of internal evaluation Dunn, Dunn2 and average Silhouette indices pointing to similarity between herbicides from different clusters in MACCS (as well other fps, results not shown) representation (Fig. 3). The unclassified Z compounds (placed in the upper right corner of the heat map in Fig. 3a) are the most structurally diverse molecules. They are structurally different mutually as well as from the rest of herbicides and thus they are unclassified. The most numerous class B (Table 1) is divided into the two relatively homogenous clusters: the 5th cluster of 49 sulfurones and sulames and the 6th cluster with 12 remaining ALS inhibitors possessing imidazoline or pyrimidinyl(thio)benzoate fragments (Fig. 1). Several herbicides with sulphonamide fragment from the other classes E, F2 and K3 are merged with the 5th cluster. The other two chemically homogenous clusters 1st and 2nd correspond to the well-known sub-groups of the ACC inhibitors of the A class—those with cyclohexanedione ring (DIMs) and those with aryloxyphenoxy-isopropionate fragment (FOPs), respectively. The five of ACC inhibitors are grouped in the 3^rd^ cluster with the subgroup of synthetic auxins O (plant hormones), on the basis of possessing common halogenated phenoxyl fragment. In difference, the PPG oxidase (chlorophyll synthesis) inhibitors of the class E are dominant in the two heterogenous clusters (cl4 and cl13/ cl4 and cl10 in Fig. 2a/b). In the cluster cl4, they are grouped with some A, C1, C3, F1, F2, K1 and K3 herbicides, while in another cluster they are put together with all ATP synthesis inhibitors from the class M.

The obtained results illustrate that herbicides from different HRAC classes share structural fragments which may direct them to the same biological activity. Such results may point to the caution in the application of the rotational anti-resistance strategy using only MoA classification systems.

In order to apply the RF model to unclassified compounds such as Z compounds and phytotoxic NPs, the AD was defined. The AD presents the region in chemical space where the model’s individual predictions are reliable. The AD boundaries were defined by the two parameters: (1) structural similarity with the training compounds and (2) the predicted RF class probability (Fig. 3c). The RF class probability has already been shown to be efficient for differentiating between reliable and unreliable predictions^36^. An RF class probability is estimated as a fraction of total number of trees which for a given compound votes for this class. It corresponds to one minus the error probability and thus provides a confidence level on the class prediction and can be used for ranking. For all training herbicides, the MoA labels were accurately predicted with the class probabilities greater than 0.6 and hence this value was taken as an AD boundary (max_rf_prob > 0.6, Fig. 3c, Table S1). For structural dissimilarity the threshold in the Jaccard index (1-TC) of 0.4 was chosen, that is an external compound should be similar to at least one of the training herbicides with a minimal TC greater than 0.6 (min_Jaccard_dist < 0.4).

The MoA class for 75.4% of the test compounds was predicted with max_rf_prob > 0.6 and for all of them the MoA was correctly predicted. In the case of the HRAC2020 set, the independent external set contains 92 herbicides (compounds assigned to the classes G, H and I were dismissed) from the HRAC_REST subset which were classified a priori on the basis of their chemical families available in the literature and online sources (Fig. 3a)^6,11–13^. Among 60 HRAC_REST compounds which lay within the AD, only ethoxyfen was predicted as A instead of E class inhibitor (Table S1) ^5^. Most of these correctly predicted but obsolete herbicides are inhibitors of photosynthesis (C1, C2, E) or fatty acid synthesis (A, K3) as well as plant growth regulators (O). Although for the majority (29) of the rest of 32 compounds the minimal TC was greater than 0.6, their class probabilities were less than the cutoff 0.6 and they were hence left unclassified. Considering Z compounds, although 55 of them are structurally similar to the training compounds with TC > 0.6, only 12 of them lye within the AD and MoA might be assigned. This illustrates that structural similarity estimated on the presence of the common structural fragment(s) in MACCS representation is not sufficient condition for conclusion upon sharing the common MoA. The more complex representation is necessary for similarity based AD definition than provided by MACCS(-like) fingerprint—one that is inherently captured by more complex models such as those provided by RF or SVM algorithms.

### Weed selectivity and application stage—descriptor and model selection

Adding descriptors which are known to describe uptake and distribution of compounds through plants, reduced the sensitivity of the MoA classification models (Table S3)^28–34^. The increase in number of FNs indicated that there are common molecular characteristics between members of different MoA classes. Herbicides are also classified according to their application stage and selectivity toward different types of weeds. The phytotoxic effectiveness greatly depends upon herbicide application timing and environmental conditions. Correct application timing maximizes weed control and limits crop injury. There are pre-emergent (here denoted as PRE) herbicides that control seedling growth of weeds and post-emergence (POST) ones which control actively growing tissue of young weeds in a way to be applied directly onto weeds and away from a crop. There are also compounds which can be applied in both regimes (BOTH). The analyzed subset of synthetic herbicides included 221 herbicides of which 49/90/82 are applied in PRE/POST/BOTH regime (Table S1)^14^. The 3-class models for the complex application stage variable built by using MACCS keys, physicochemical and/or simple molecular features of compounds without considering environmental variables, had, in general, lower predictive power (test set: accuracy ~ 0.62, kappa ~ 0.40) than the predictive models for MOAs (Table 2) and weed selectivity (Table 3). Hence, we did not pursue further model analysis and interpretation.

**Table 3:** Comparison of performance metrics on the test set of 3-class RF and SVM models built for prediction of BL, G or NS weed selectivity of herbicides in terms of subset of nine simple molecular and physicochemical descriptors including lipophilicity coefficient logP or 141 MACCS keys^a^.

RF /SVM^b^	Per classes				
9 descriptors with logP	Sensitivity	Specificity	Precision	F1	Balanced Accuracy
Class: BL	0.944/0.917	0.690/0.690	0.791/0.786	0.861/0.846	0.817/0.803
Class: G	0.739/0.696	0.952/0.929	0.895/0.842	0.810/0.762	0.846/0.812
Class: NS	0.500/0.667	1.000/1.000	1.000/1.000	0.667/0.800	0.750/0.883

**141 MACCS**					
Class: BL	1.000/1.000	0.793/0.828	0.857/0.878	0.923/0.935	0.897/0.914
Class: G	0.783/0.826	1.000/1.000	1.000/1.000	0.878/0.905	0.891/0.913
Class: NS	0.833/0.833	1.000/1.000	1.000/1.000	0.909/0.909	0.917/0.917

^a^The nine descriptors are logDiff, logSw, Shapeindex, Cat, sp3At, TPSA, HBA, HBD plus logP.

^b^The RF and SVM models with 9 descriptors including log P/141 MACCS keys correspond to the models **1** and **7**/**3** and **9**, respectively, in Table S6. The models were trained and applied with using tuned hyperparameters’ values (Figures S2–S4).

Herbicides may be divided into the three classes with regard to weed selectivity: herbicides which act selectively against broadleaf (BL) or grass (G) weeds and those which are non-selective (NS) and act on broad spectrum of weeds^40^. The BL or G herbicides clear away only certain weeds by acting on processes that are more important for those types of weeds, while the NS herbicides act on processes that are important in all plants. Although the weed resistance is observed for herbicides regardless of their weed selectivity class, the rotational change of herbicides with different selectivity may reduce weed resistance caused by change in herbicide translocation profile ^8^. In the data subset of 332 herbicides, 181 BL selective herbicides are from MoA classes C1, C2 and E associated with the photosynthesis inhibition and the class O of growth regulators. The 118 G selective herbicides are from the classes A, K1, K3 and N and are mostly inhibitors of fatty acid synthesis. The most of 33 collected NS herbicides are mainly from the classes B, D and P. The most prone to weed resistance are inhibitors from the classes B, C1-C3, A and G^5^.

The 3-class RF models were built by dividing 332-data set into 267 training and 65 test compounds represented by MACCS keys and more than 160 other molecular properties. By employing the later set of descriptors, the nine conceptually clear and whole molecular features were identified among most important and efficient for herbicide differentiation according to weed selectivity (Table 3). Adding or using other descriptors did not change predictive power of models significantly. These are partition (logP) or distribution (logD at pH 7.4) coefficient, native solubility Sw in pure water at 25 °C (transformed to log(Sw/mol L^-1^)), diffusion coefficient in water (Hayduk-Laudie formula, log(Diff × 10^–5^/ (cm^2^/s)), TPSA as well as numbers HBA and HBD all calculated by ADMET Predictor^27^, as well as ShapeIndex (spherical < 0.5 < linear) and numbers of sp^3^-hybridized (sp3At) and all carbon (Cat) atoms within molecule calculated by DataWarrior^26^.

**Figure 4:**
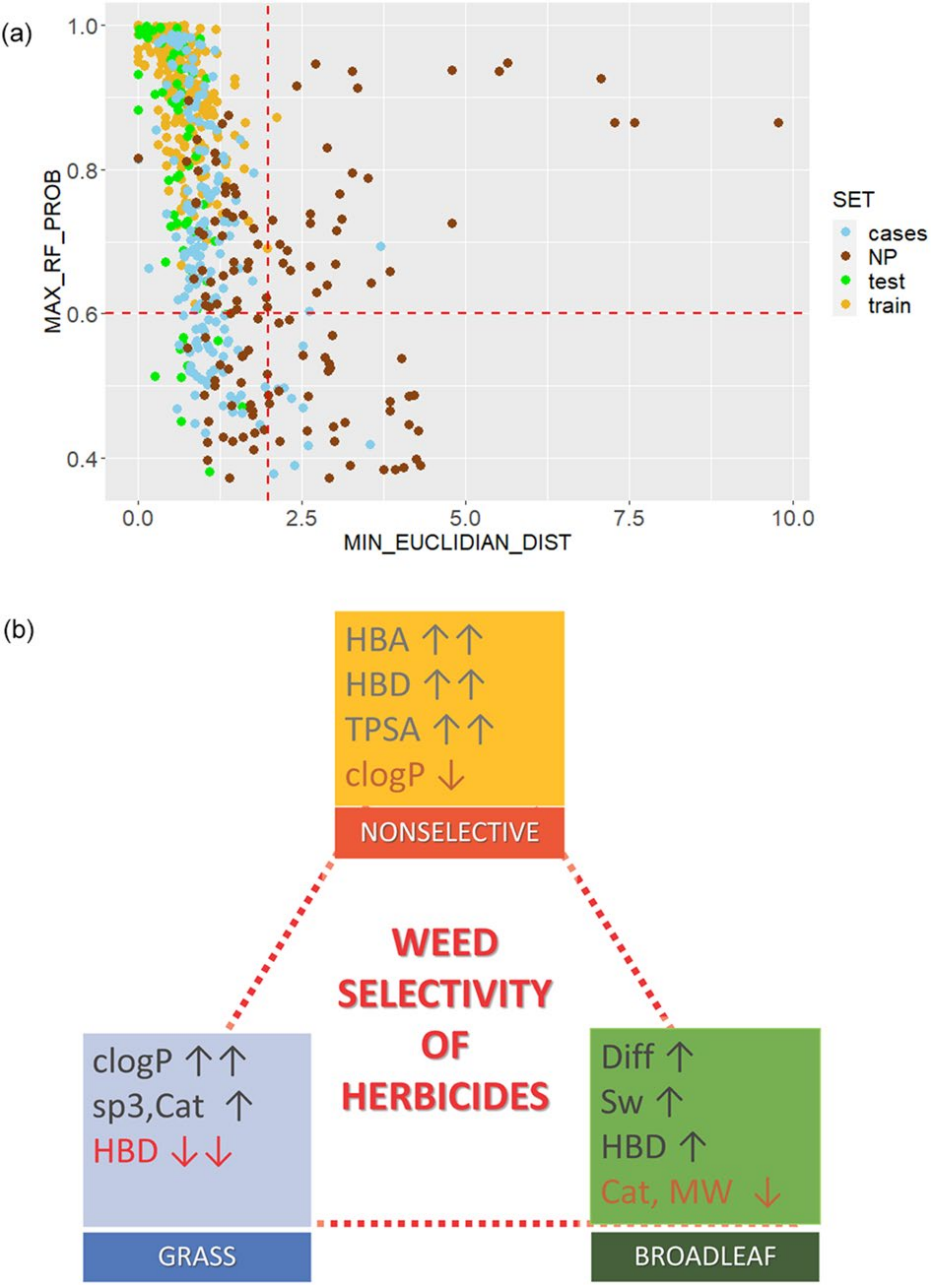
(**a**) The AD for the RF weed selectivity model (**1** in Tables 3 and S6). Given a compound, the prediction can be considered credible for the class probability above 0.6 and the Euclidian distance less than 2.0. (**b**) The most distinguishing molecular features of the broad-leaved or grass selective and non-selective herbicides.

Among explored ML classifiers the most competitive were RF and SVM models (Fig. 1e, Table 3, Table S6). The RF and SVM predictions differ mutually for one/three test compounds and 36/24 case compounds described in terms of MACCS fp /nine whole molecular features including logP without taking AD criteria into regard. Although classification of synthetic herbicides into BL, G and NS classes was somewhat better in terms of MACCS fp (Table 3), we decided to promote the set of whole molecular descriptors. The later descriptors provide simple and meaningful interpretation to the potential end users including chemists interested in discovery and development of not only novel herbicides but also molecular probes for investigation of biological processes in plants. Additionally, in comparison with the models built in terms of MACCS fp keys, the models built in terms of physicochemical and whole molecular descriptors are more general and may not be limited to structurally similar compounds as it is demonstrated by comparison of the ADs in Fig. 3c vs Fig. 4a. The use of either logP or logD did not impact predictive power of the RF models considerably (Table S6). Since logP coefficients are more readily calculated, the further analysis is focused on the RF model with logP.

### Weed selectivity—physicochemical space analysis

The AD for the RF model (**1** in Tables 3 and S6) is defined by the use of its class probability outputs and Euclidean similarity with the training compounds in the physicochemical space spanned by the nine descriptors (Fig. 4a). All training compounds were predicted with the class probability above 0.6. The RF model predicts correctly weed selectivity for more than 3/4 of 65 test synthetic herbicides using the thresholds of 0.6 for class probability and 2.0 for Euclidian distance (Fig. 4a). The half of the rest of the test compounds was either left unclassified (class probability < 0.6) or were wrongly assigned in spite of their similarity with the training compounds in the physicochemical space.

Considering 177 external case compounds, 135 were within the AD and for them weed selectivity was assigned using the probability cutoff of 0.6 (Table S1, Fig. 4a). Most of these synthetic herbicides were predicted to be BL by all classifiers (Table S1).

The nine physicochemical and simple molecular properties are, in general, associated with uptake and translocation of compounds through plants^41,42^. However, this observed dependence of the weed type selectivity may also be related to the specific sub- cellular/plastid location of target proteins (pathways) and/or to different characteristics of binding sites of herbicides on targets. As compared with the BL and G selective compounds, the NS herbicides are more polar molecules possessing larger polar surfaces TPSA and more HBA (> 5) and HBD (mostly 2) heteroatoms and hence they are more hydrophilic (smaller logP/logD values and more soluble in water) (Figs. 4b and S5). In opposite, the G selective herbicides are molecules with the smallest number of HBD atoms and the smallest relative polar surface. Majority of BL herbicides have one HBD atom. While most of the broad-spectrum NS herbicides have logP lower than 2, most of selective herbicides particularly of the G type has logP greater than 3.0. The BL selective herbicides have the smallest number of sp3 hybridized atoms, molecular weight and molecular volume what may be reflected in their distinguishing diffusion and distribution properties in comparison with herbicides from the other two selectivity classes^43^.

### Assessing the potential of phytotoxic natural products

Natural products are a treasured source for novel biologically active compounds, including those with phytotoxic effect^15,18^. So far NPs have had a relatively small impact on the discovery and development of novel herbicides as compared with insecticides and fungicides. Less than 10% of active ingredients registrations for weed management have been of natural origin^16^. However, in ten of the HRAC classes either a NP, a semisynthetic derivative or synthetic herbicide inspired by a natural scaffold are present^18^. Importantly, most of NPs have different modes of phytotoxic activity than synthetic organic herbicides^16,19,21^.

**Figure 5:**
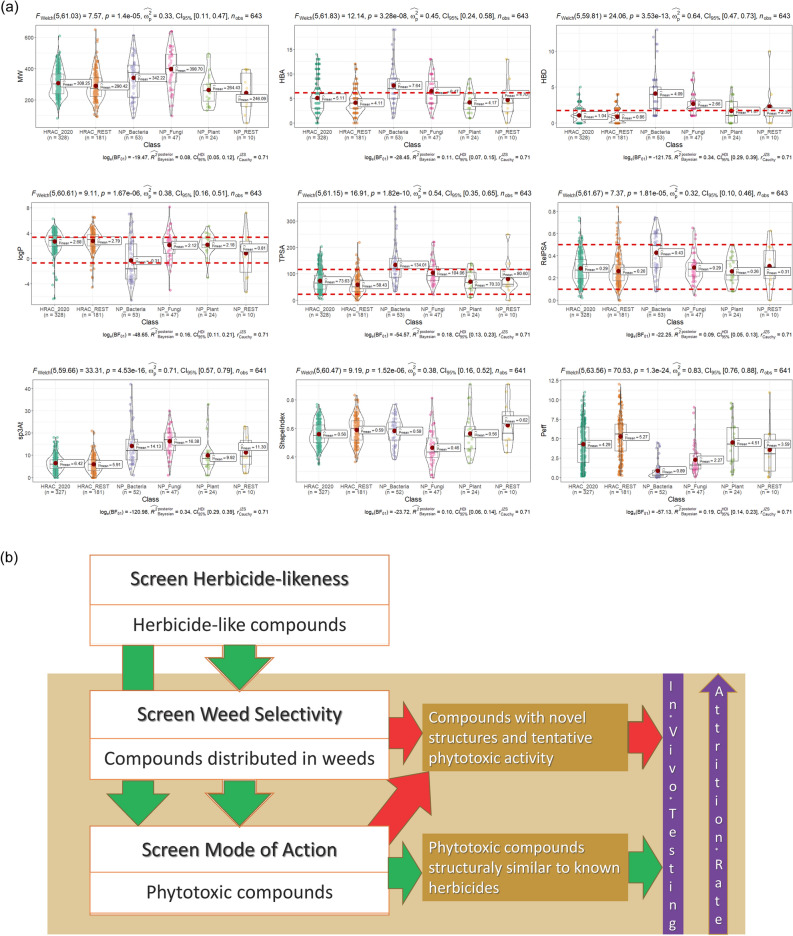
(**a**) The comparison of six subgroups of phytotoxic molecules according to selected molecular properties. Herbicide-like boundaries (Table 4) are denoted by red dash lines. (**b**) Virtual screening platform proposed for preselecting phytotoxic compounds. Its proof-of-concept should be carried out by in vivo testing.

The data set of 131 phytotoxic NPs, with MW less than 650, was collected from the literature^15,16,19^. They are mainly of bacterial (39.6%), fungal (35.1%) or plant (17.9%) origin (Table S2). Although coming from different sources, these natural compounds are structurally more similar mutually than to the synthetic herbicides (Fig. 3b). Since phytotoxic NPs are structurally different, they fall outside the ADs of the models based on the MACCS structural keys of the synthetic herbicides (Fig. 3c). In comparison, more than half of NPs are similar to the training compounds within space defined by the nine descriptors, having Euclidian distance less than 2.0 (Fig. 4a, Table S2). However, only 1/3 of the whole NP set fall within the AD RF model. This analysis indicated that NPs may differ from synthetic herbicides not only in structural space and MoAs, but also in space of the physicochemical and simple molecular features which are often associated with uptake and translocation properties (Fig. 5a and Figure S6)^28–34^.

### Herbicide-like properties

For synthetic herbicides distributions of physicochemical and simple molecular properties have already been reported^28–34^. These simple molecular properties and physicochemical features largely influence the mass distribution of herbicides across plants and plant cell compartments and hence may be applied for characterizing herbicide-likeness of compounds^41,42^. The phytotoxic effect of a herbicide largely depends upon its translocation through plants to its site of action analogously as pharmacological effects of drugs are considerably influenced by their absorption and distribution throughout the human body^44^. Drug-likeness filters are commonly used in early drug discovery process to eliminate compounds out of the sets aimed for biological activity screening. In analogous way, herbicide-likeness features may be used as a first-pass filter for eliminating compounds from the analyzed compound data sets and libraries which are less probable to show biological activity in weeds. The proposed herbicide-like features obtained by analyzing the extended set of 509 synthetic organic herbicides with MW less than 650 Da, are listed in Table 4. They were applied on the data set of NPs.

**Table 4:** Herbicide-like chemical space defined in terms of common molecular descriptors (Fig. 5).

Descriptor	Range	% of 509 synthetic herbicides	% of 131 NPs
HBD (OH/NH)	≤ 2	95	51.9
HBA (O/N)	≤ 6; ≤ 7	66.7; 80.0	58.8; 65.6
clogP^a^	0.5 < clogP ≤ 3.5;0.5 < clogP ≤ 4.5	66.7; 80.0	47.3; 53.4
TPSA	20 Å^2^ < TPSA≤ 120 Å^2^	80	63.4
Relative PSA	0.1 <RelPSA ≤ 0.5	80	81.7
Net charge^b^	≤ 0	95	65.6

^a^Regardless logP values were calculated by ADMET Predictor or DataWarrior.

^b^More than 95% of synthetic organic herbicides are either neutral molecules (around 2/3) or anions (30%) (Figure S7).

Phytotoxic molecules produced by plants are found to be the most similar to the synthetic herbicides both in structural and physicochemical spaces (Fig. 5a). In difference, fungal and particularly bacterial NPs vary in the physicochemical space from the rest of studied compounds (Figures S6 and S7). They are richer in H-bond interacting atoms similarly as many other types of NPs^45^. The bacterial phytotoxic compounds are relatively more polar, hydrophilic and charged molecular species. The fungal products have more sp3-hybridized atoms and are also more spherical compounds what may imply their different translocation capacity and features. The most of bacterial and fungal phytotoxic compounds were estimated to have lower permeation rates (Peff (cm/s x 10^4^) in Fig. 5a) across lipophilic membranes as compared with the plant NPs and synthetic organic herbicides. The lower membrane permeability is generally associated with compounds having lower lipophilicity and larger number of H-bond interacting atoms, particularly larger number of HBD atoms and may also be caused by the membrane retention^42,45^. However, the uptake and translocation of a small dissolved phytotoxic NPs can be determined not only by their passive permeation across membranes, but also by the active translocation by transport proteins^8^. The translocation propensity of bacterial and some fungal compounds can also be affected by the presence of ionized carboxyl group(s)^46^.

### In silico screening platform

The comprehensive modelling carried out on the set of synthetic herbicides and application of the models and herbicide-likeness filter on phytotoxic NPs encouraged us to propose the in silico screening platform which can be applied on any set /library of compounds for characterization of their herbicide-likeness and possibly phytotoxic ways of action (Fig. 5b). Considering the data set of 131 NPs, 81 molecules satisfy 4 or more herbicide-likeness criteria (Table 4), and 35 of them lay within the AD of the RF weed selectivity model (Fig. 4a), while all are outside the AD of the MoA and other models built in terms of specific structural fp keys. This result suggests further experimental studies that might reveal new MoAs for these compounds, which in turn may lead to new herbicides, potentially also adding more robustness to the current rotational strategies for minimizing weed resistance, based on available classes of herbicides.

## Conclusions

There are two main ways to minimize weed resistance, the application of herbicides according to the rotation strategy which is well-accepted by the end users and to discover and develop novel phytotoxic compounds. The developed predictive classifiers to a large extent confirm MoAs assignation for the HRAC herbicides based on structural similarity and additionally enables MoA assignment for herbicides, mainly obsolete due to their side effects and thus lying outside the HRAC list. However, the performed modelling points out limitations of using only structural similarity for MoA classification and further for selection of herbicides for rotation strategy. The conducted ML modelling of weed selectivity reveals that it is largely determined by simple molecular and physicochemical features which also influence uptake and distribution of small molecules through plants. Since similarity in uptake and translocation properties of herbicides may lead to the similar mechanisms of induction of weed resistance, the weed selectivity categorization is suggested as an additional rotational criterion.

The additional output of the study is the proposal of in silico stepwise screening platform for detecting herbicide-like molecules with selectivity for weed types and possibly with pre-specified mode of action, from any chemical library or database (Fig. 5b). Application of the platform to the data set of pyhtotoxic natural products reveals that they lie outside the space of synthetic herbicides considering not only molecular structure, but also physicochemical properties guiding weed selectivity. Therefore, natural products might represent worthy source of novel phytotoxic scaffolds with new/different modes of action, thus contributing to more effective and weed-resistance robust use of herbicides.

The proposed herbicide-likeness and screening cascade can be used for prioritization of the in vivo experiments.

## Supplementary Information

Supplementary Table S1.

Supplementary Table S2.

Supplementary  Tables S3-S6 and Figures S1-S7.

## Data Availability

The R scripts and data sets for model performance are available at GitHub (https://github.com/mlkr-rbi/Herbicide-Classification.git). Data sets analyzed and/or generated during the current study are available in Supplementary information.
