# Supplementary material for: Comprehensive machine learning based study of the chemical space of herbicides
Source: Sci Rep. 2021 Jun 1;11:11479. doi: 10.1038/s41598-021-90690-w (PMC8169684; doi:10.1038/s41598-021-90690-w)
Supplement: Supplementary file 3 — Supplementary Tables S3-S6 and Figures S1-S7. [file 41598_2021_90690_MOESM3_ESM.pdf]

Supplementary Information

Comprehensive machine learning based study of the chemical space of herbicides

Davor Oršolić,[1] Vesna Pehar,[2] Tomislav Šmuc[1] & Višnja Stepanić[1*]

[1] Laboratory for Machine Learning and Knowledge Representation, Division of Electronics, Rudjer Bošković Institute, Bijenička 54, HR-10002, Zagreb, Croatia

[2] Croatian Defense Academy "Dr. Franjo Tuđman", Ilica 256b, 1000 Zagreb, Croatia

* Corresponding author: V.S.: tel, +38514571356; e-mail, stepanic@irb.hr

ORCID ID: Davor Oršolić, 0000-0002-5385-1031; Vesna Pehar 0000-0002-9652-0144; Tomislav Šmuc 0000-0002-9185-9384; Visnja Stepanic, 0000-0001-9518-4153

1) DESCRIPTOR TESTING

Table S3. Validation statistics for the multi-class RF models (ntree=500) built by using extended herbicide set (HRAC2020+HRAC_REST, Table S1) by using subsets of various types of descriptors for predicting modes of action of herbicides.[a]

SET	1: MACCS[b]	2: Constitutional & electronical[c]	3: 2 + BCUTs	4: 1+ 3	5: 1 + AP
**TRAINING**					
**Accuracy**	0.854/0.854	0.792	0.806	0.833	0.824
**κ-value**	0.839/0.839	0.769	0.786	0.815	0.806
**TEST**					
**Accuracy**	0.872/0.895	0.808	0.821	0.846	0.846
**κ-value**	0.859/0.883	0.786	0.801	0.831	0.831
**Av Sensitivity**	0.728/0.821	0.597	0.589	0.709	0.709
**Av Specificity**	0.993/0.993	0.988	0.988	0.991	0.991

[a] In the model **5**, the MACCS keys from the model **1** was combined with 33 descriptors calculates by the software ADMET Predictor (AP):molecular properties (MW, molecular volume, HBA,HBD, TPSA, topological indices related to the molecular shape like T_Rgrav, T_Radb, T_Radc, T_Rads, T_HydroR, T_MIRxx, T_MIRyy) and physicochemical descriptors (logP, logD, logPeff (Peff – estimated passive membrane permeability), logSw (Sw-estimated water solubility)) [Lowless et al., 2016]. [b] For the purpose of comparison the 1st /2nd value of each statistics corresponds to modelling on the extended/HRAC2020 data set (Table 1).

2) HYPERPARAMETER TUNING FOR THE FOUR CLASSIFIERS EXPLORED

Mode of action classes (16-class, with MACCS)

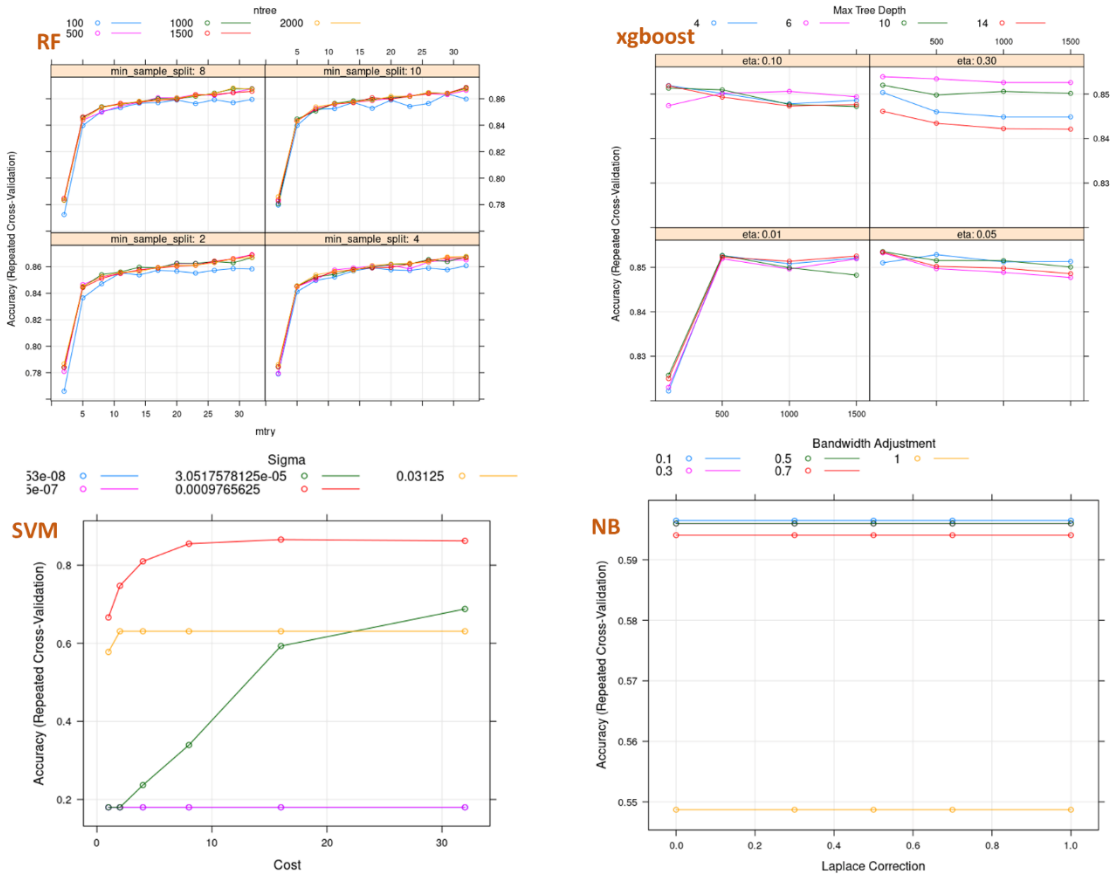

Figure S1. Hyperparameter tuning for the four ML classifiers for predictions 16 HRAC MoA classes in terms of MACCS keys, was performed by *tuneGrid* of the R package *caret*. For RF and NB classifiers, parameter tuning was done by utilization the packages *randomForest* and *klaR*, respectively. For SVM we used RBF kernel (linear SVM turned out to have inferior performance to kernel based SVM) and performed grid search over the cost parameter and the RBF kernel parameter sigma. NB algorithm achieves significantly lower accuracy, which is expected due to inability to model variable interactions and assumption of independence between variables.

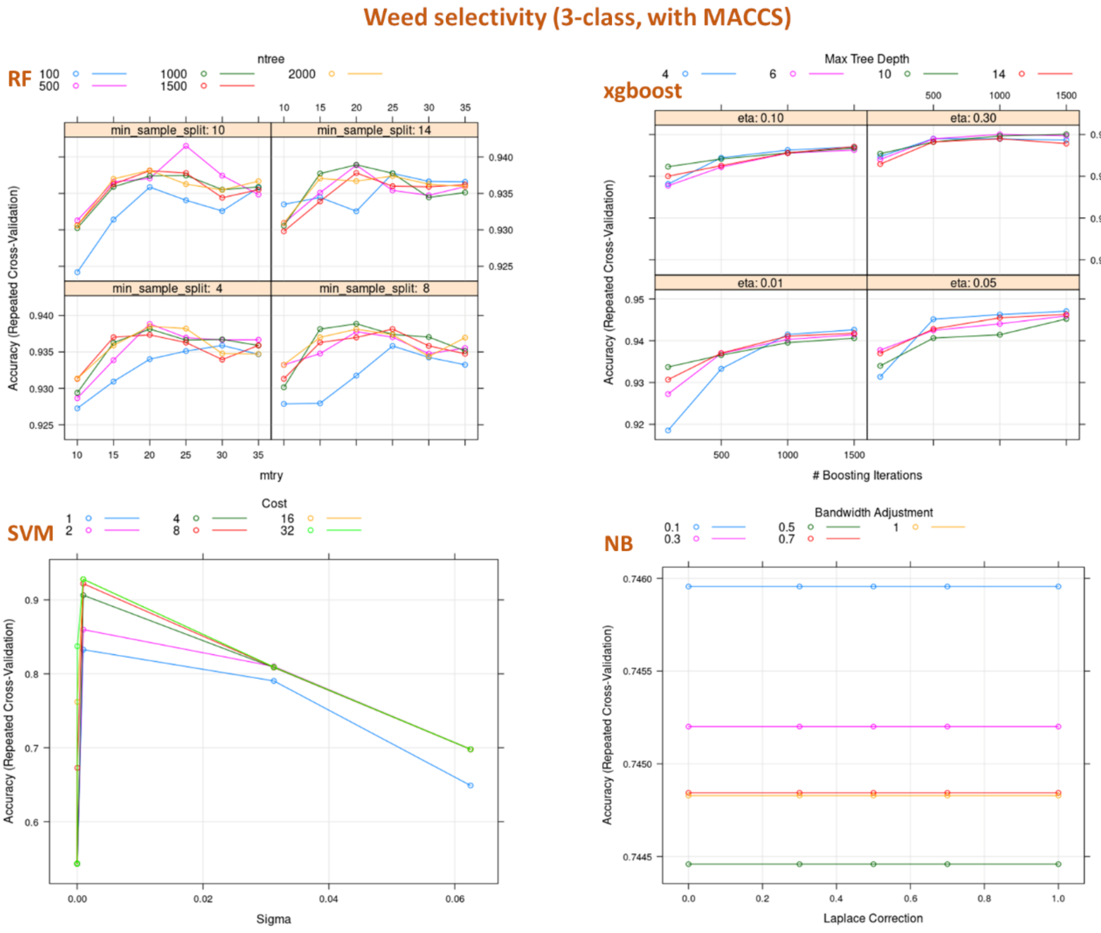

Figure S2. Hyperparameter tuning in the four ML models built in terms of structural MACCS keys for predictions three weed selectivity classes, was done in analogous way as for the 16-class MoA classifier. NB algorithm achieves significantly lower accuracy, which is expected due to inability to model variable interactions and assumption of independence between variables.

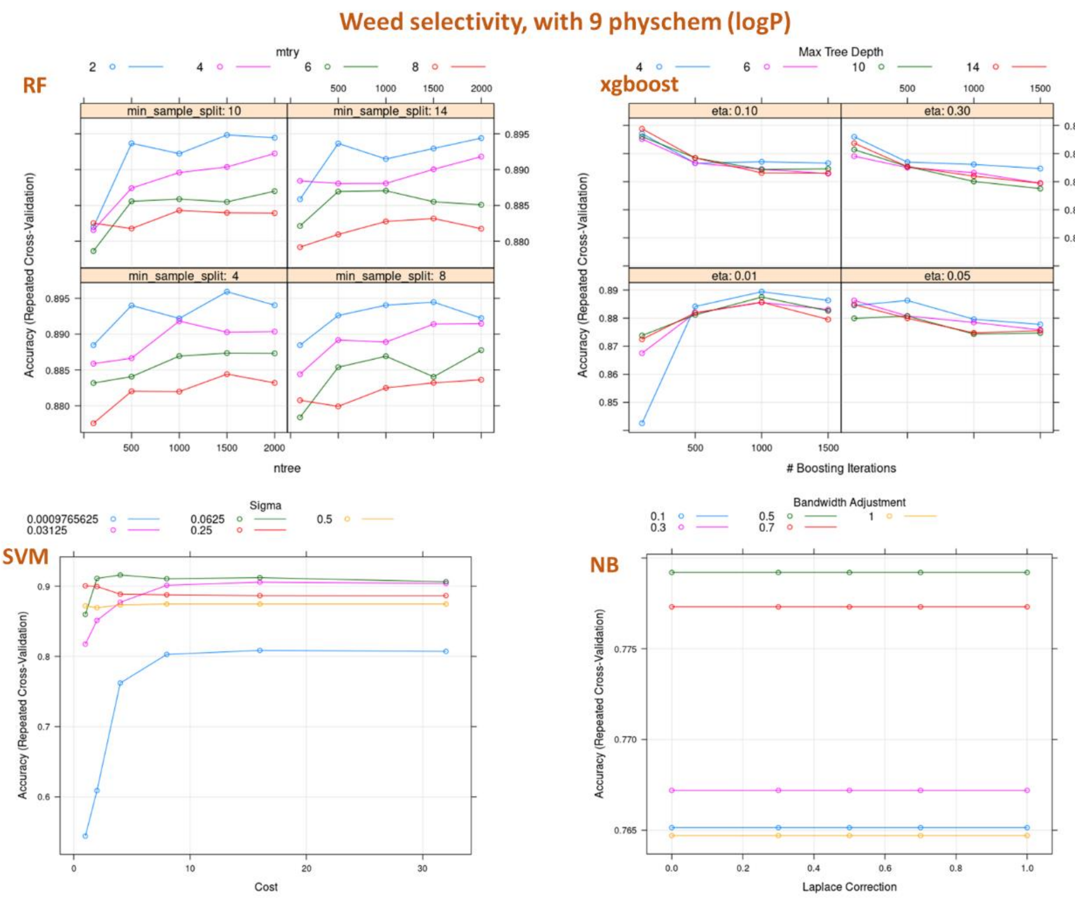

Figure S3. Hyperparameter tuning in the four ML models built in terms of whole molecular features including lipophilicity coeficient logP, for predictions three weed selectivity classes, was carried out in analogous way as for other classifiers. NB algorithm achieves significantly lower accuracy, which is expected due to inability to model variable interactions and assumption of independence between variables.

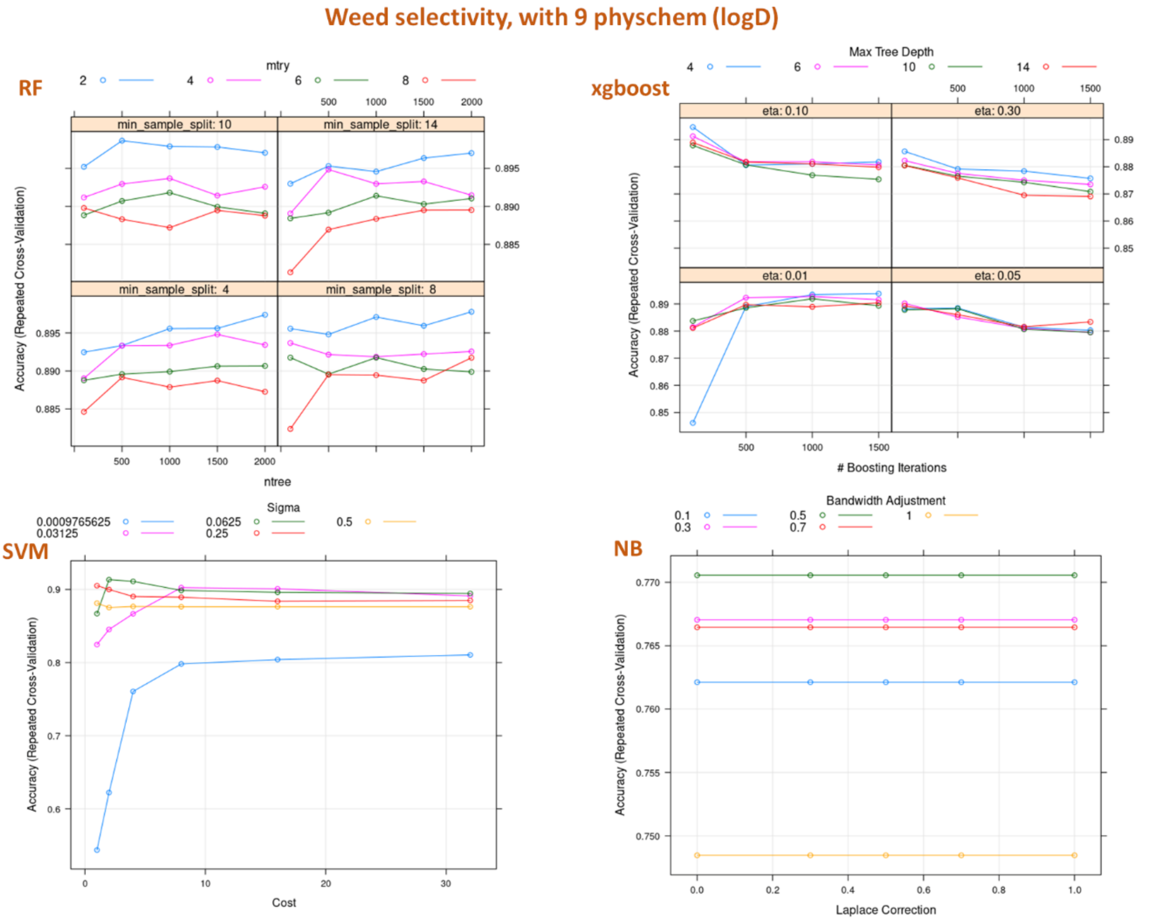

Figure S4. Hyperparameter tuning in the four ML models built in terms of whole molecular features including lipophilicity coeficient logD@pH 7.4, for predictions three weed selectivity classes, was carried out in analogous way as for other classifiers. NB algorithm achieves significantly lower accuracy, which is expected due to inability to model variable interactions and assumption of independence between variables.

Table S4. Optimal hyperparameters for the four classifiers employed for predictions HRAC mode of action (MoA) and weed selectivity classes.

	Random Forest			SVM		NB		
**Model**	**mtry**	**ntree**	**min_sample_split**	**sigma**	**C**	**fL**	**adjust**	**usekernel**
**HRAC MoA**	32	500	2	0.000976563	16	0	TRUE	0.1
**Sel - LogD**	2	500	10	0.0625	2	0	TRUE	0.5
**Sel - LogP**	2	1500	4	0.0625	4	0	TRUE	0.5
**Sel - MACCS**	25	500	10	0.000976563	32	0	TRUE	0.1

	**XGBoost** (eXtreme Gradient Boosting)						
**Model**	**nrounds**	**max_depth**	**eta**	**gamma**	**colsample_bytree**	**min_child_weight**	**subsample**
**HRAC MoA**	100	6	0.3	0	0.5	1	1
**Sel - LogD**	100	4	0.1	0	0.5	1	1
**Sel - LogP**	1000	4	0.1	0	0.5	1	1
**Sel - MACCS**	1500	10	0.3	0	0.5	1	1

3) MODELS'S CHARACTERISTICS

Table S5a. The top 20 ranking MACCS variables as determined by the *randomForest* package based on mean decrease of accuracy, using optimized RF parameters, for the two problems: HRAC and weed selectivity. The two models share eight variables among 20 top important ones, which indicated that some of different HRAC classes (structurally different compounds) share same weed selectivity label. *Random Forest* variable importance procedure was used as it is superior to the standard *caret* varImp procedure which for the SVM (also NB) uses model independent scheme, which does not take into account variable interactions when determining individual variable importance. Meaning of MACCS key is taken from https://github.com/rdkit/rdkit/blob/master/rdkit/Chem/MACCSkeys.py

HRAC	MACCS key meaning	Weed selectivity	MACCS key meaning
**V77**	NAN	V87	X!A$A
**V151**	NH	V43	QHAQH
**V37**	NC(O)N	**V133**	A$A!N
**V133**	A$A!N	**V77**	NAN
**V140**	O > 3 (&...)	**V140**	O > 3 (&...)
V139	OH	**V103**	CL
**V98**	QAAAAA@1	V131	QH > 1
V132	OACH2A	V131	QH > 1
V33	NS	**V145**	6M ring > 1
**V25**	NC(N)N	**V151**	NH
V126	A!O!A	V108	CH3AAACH2A
**V145**	6M ring > 1	V128	ACH2AAACH2A
V123	OCO	V122	AN(A)A
V127	A$A!O > 1 (&...)	V106	QA(Q)Q
V143	A$A!O	V107	XA(A)A
**V146**	O > 2	V142	N > 1
V42	F	V136	O=A>1
**V103**	CL	V120	Heterocyclic atom > 1 (&...)
V157	C-O	**V98**	QAAAAA@1
V148	AQ(A)A	**V146**	O > 2

Table S5b. Variables important for the two studied classification problems as determined by the R packages *caret* using ROC curve analysis.

MoA		Weed selectivity	
HRAC_RF	HRAC_SVM	Sel_RF	Sel_SVM
V106	V106	V102	V102
V110	V110	V103	V103
V112	V112	V107	V107
V117	V117	V108	V108
V120	V120	V111	V111
V121	V121	V112	V112
V126	V123	V120	V120
V127	V126	V121	V121
V130	V127	V130	V130
V131	V130	V131	V131
V133	V131	V134	V134
V136	V133	V136	V136
V140	V136	V137	V137
V142	V140	V138	V138
V143	V142	V140	V140
V145	V143	V142	V142
V146	V145	V145	V145
V148	V146	V151	V151
V150	V148	V25	V25
V151	V151	V32	V32
V152	V152	V33	V33
V154	V157	V37	V37
V157	V159	V43	V43
V159	V25	V51	V51
V25	V32	V55	V55
V32	V33	V58	V58
V33	V37	V60	V60
V37	V43	V61	V61
V43	V51	V64	V64
V51	V55	V67	V67
V55	V58	V69	V69
V58	V60	V73	V73
V60	V61	V77	V77
V61	V64	V78	V87
V64	V67	V87	V93
V67	V72	V93	V98

V72	V73	V98	
V73	V77		
V77	V80		
V80	V81		
V81	V88		
V88	V93		
V93	V98		
V98			

Table S6. Comparison of overall accuracy and kappa values of 3-class models for predicting weed selectivity, built by four ML algorithms in terms of three descriptor sets (logP/logD - logDiff, logSw, Shapeindex, Cat, sp3At, TPSA, HBA, HBD plus logP or logD; maccs – 141 MACCS keys).

MODEL	**1** RF_logP	**2** RF_logD	**3** RF_maccs	**4** xgboost_logP	**5** xgboost_logD	**6** xgboost_maccs
Accuracy	0.831	0.815	0.908	0.831	0.831	0.892
Kappa	0.677	0.684	0.828	0.684	0.681	0.802
MODEL	**7** SVM_logP	**8** SVM_logD	**9** SVM_maccs	**10** NB_logP	**11** NB_logD	**12** NB_maccs
Accuracy	0.815	0.815	0.923	0.800	0.754	0.754
Kappa	0.654	0.654	0.858	0.626	0.540	0.494

4) PCA ANALYSIS AND PHYSICOCHEMICAL PROPERTIES FOR WEED SELECTIVITY

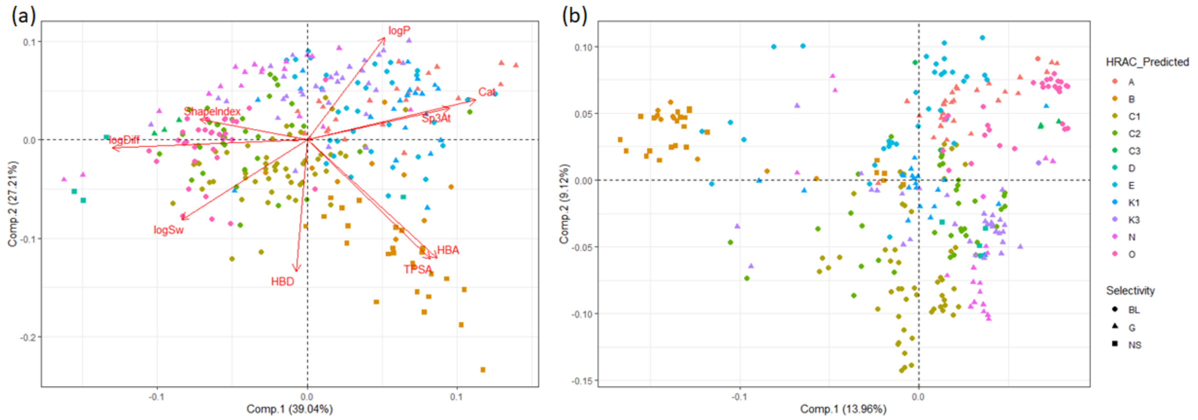

Figure S5. (a) The PCA biplot for 299 herbicides from largest MoA classes represented by nine physicochemical and simple molecular features. (b) The PCA score plot for the same subset of herbicides described by the 141 MACCS keys. Compounds are coloured according to their HRAC MoA class (Table 1), while shape is determined by their weed selectivity (BL- broadleaf, G- grass, NS – non-selective).

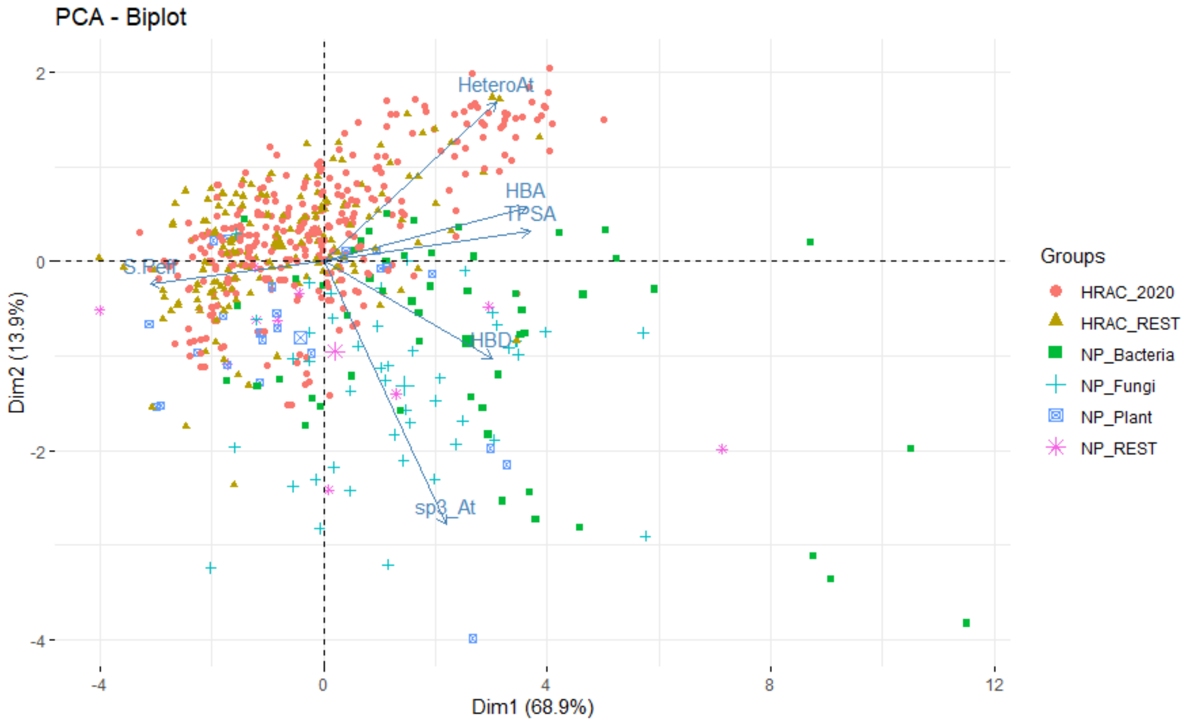

Figure S6. The PCA analysis of considered phytotoxic compounds described by simply calculated and conceptually straightforward molecular features. Phytotoxic natural products (NP) differ from synthetic herbicides (HRAC_2020 and HRAC_TEST) an well as mutually in dependance of their source.

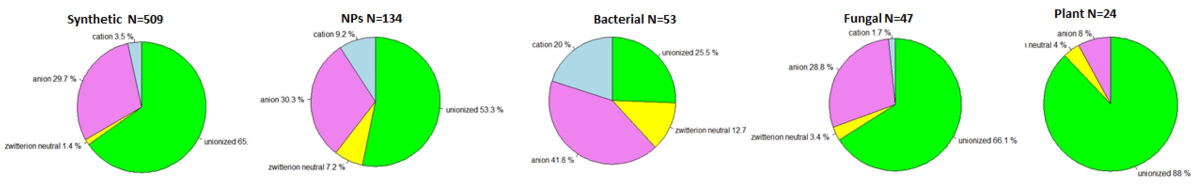

Figure S7. Fractions of synthetic and natural phytotoxic compounds which are neutral (noninonized and neutral zwitter ions), with a net negative (anion) or positive (cation) electric charge.
